# Proceedings of the Georgia Medical Society

**Published:** 1880-04

**Authors:** 


PROCEEDINGS OF THE GEORGIA MEDICAL SOCIETY.—We invite the special attention of our readers to the proceedings of a recent meeting of the Georgia Medical Society, of Savannah, in another part of this issue, in regard to certain proposed legislation now before Congress. And like the friends and brethren whose names are appended to that protest, we unhesitatingly add our disapproval of any action by our senators and representatives looking to the destruction of the autonomy of the States of this Union. This country *is not* a nation in any proper signification of the term, and when Congress attempts to make it so by overriding and trampling upon the rights of the individual States, even doctors, who usually ignore political questions and actions, should not keep silent, lest the very dust of the founders of the Republic should cry aloud from their graves against such iniquity! We never held office not appertaining legitimately to our profession, and cannot be regarded in any sense a politician. But as a lineal descendant of gentlemen who participated in the struggles against the mother-country for the liberation of the original colonies, and the maintenance in the subsequent conflict with her of the honor and dignity of the United States, we feel in duty bound to enter our decided and solemn protest against *any* legislation tending to, or even looking in a remote degree toward empire, or the unification of the States and Territories of this country.

